# Doxorubicin–Cyclophosphamide Protocol in Dogs with Splenic Haemangiosarcoma and Haemoabdomen: A Retrospective Case Series

**DOI:** 10.3390/vetsci12111053

**Published:** 2025-11-02

**Authors:** Noemí del Castillo, Manuel Fuertes-Recuero, Elisabetta De Angelis, Claudia de la Riva, Cristina García, Noemí Rayón, Sandra Márquez, Gustavo Ortiz-Díez

**Affiliations:** 1 Deparment of Oncology, Universidad Alfonso X el Sabio (UAX), Avenida de la Universidad 1, Villanueva de la Cañada, 28691 Madrid, Spain; 2 Centro Veterinario Surbatan, C/Cebreros, Villanueva de la Cañada, 28011 Madrid, Spain; 3 Complutense Veterinary Teaching Hospital, Complutense University of Madrid, Avda. Puerta de Hierro s/n, Villanueva de la Cañada, 28040 Madrid, Spain; gusortiz@ucm.es; 4 Department of Physiology, Veterinary Medicine School, Complutense University of Madrid, Avda. Puerta de Hierro s/n, Villanueva de la Cañada, 28040 Madrid, Spain; 5 Department of Animal Medicine and Surgery, Veterinary Medicine School, Complutense University of Madrid, Avda. Puerta de Hierro s/n, Villanueva de la Cañada, 28040 Madrid, Spain

**Keywords:** canine, splenic haemangiosarcoma, chemotherapy, haemoabdomen

## Abstract

This retrospective case series describes 21 dogs with stage II splenic haemangiosarcoma (HSA) and spontaneous haemoabdomen treated with splenectomy followed by a doxorubicin–cyclophosphamide protocol. Median overall survival was 92 days, with longer survival observed in dogs completing three or more chemotherapy cycles. The regimen was well tolerated, with low haematological and gastrointestinal toxicity. Maintenance metronomic chemotherapy was administered in a small subset, and it was associated with prolonged survival. While the sample size is modest, the homogeneous and clinically specific cohort provides hypothesis-generating observations on feasibility, tolerability, and time-aware associations that warrant cautious interpretation and prospective confirmation. No clinical or laboratory variable at diagnosis was independently associated with outcome. While limited by retrospective design and small sample size, this study provides focused data on a specific clinical presentation and supports the feasibility of structured adjuvant treatment in dogs with splenic HSA and haemoabdomen.

## Simple Summary

Many dogs develop a fast-growing cancer of the spleen that can rupture and cause internal bleeding in the abdomen. This emergency often requires the spleen to be removed, but it is unclear which follow-up treatments help dogs to live longer. We reviewed the medical records of 21 dogs that underwent splenectomy for this cancer and received a planned course of two commonly used chemotherapy drugs (doxorubicin and cyclophosphamide). We investigated how long the dogs survived, how well they tolerated the treatment and whether completing more of the planned cycles made a difference. The median survival time after surgery was approximately three months (92 days). Dogs that completed three or more cycles survived for around 200 days, whereas those that received fewer cycles survived for around 55 days. Side effects were generally mild and serious problems were rare. Some dogs also received continuous, very-low-dose daily chemotherapy later on, and they lived longer. However, the numbers were too small to draw firm conclusions. No single test result at diagnosis predicted outcome. These findings suggest that a structured, well-tolerated chemotherapy plan following surgery could help some dogs with this emergency cancer to live longer. Further research is needed to confirm the most effective approach.

## 1. Introduction

Splenic haemangiosarcoma (HSA) is a frequent diagnosis in canine oncology and a leading cause of spontaneous haemoabdomen requiring emergency splenectomy [1,2]. Biologically, HSA is an aggressive endothelial neoplasm with occult micrometastases at diagnosis, and early dissemination to the liver, omentum/mesentery, lungs or other organs; consequently, surgery alone rarely provides durable control [3,4,5]. Despite its clinical relevance, dogs presenting with intra-abdominal haemorrhage remain under-represented in published cohorts. Many series combine splenic HSA with other anatomical variants or do not distinguish ruptured from non-ruptured tumours [6,7], masking the specific prognostic impact of haemoperitoneum.

From a staging and perioperative perspective, tumour rupture typically denotes stage II disease and often necessitates stabilisation, transfusion support, and the timely initiation of adjuvant therapy [4,8]. These constraints are especially salient in haemoperitoneum, where immediate postoperative tolerance to intensive cytotoxic regimens may be limited [1,9].

Following splenectomy, adjuvant doxorubicin-based chemotherapy is widely recommended [8,10]. The vincristine–doxorubicin–cyclophosphamide (VAC) protocol has traditionally served as the reference regimen, but its adoption is constrained by substantial myelosuppression and gastrointestinal toxicity [3,9]. In response, alternative strategies have been explored, including doxorubicin–cyclophosphamide protocol [4], single-agent doxorubicin schedules [9] doxorubicin–dacarbazine combinations [10], carboplatin-based approaches [11], and optimisation of adjuvant timing [11]. Among simplified regimens, the doxorubicin–cyclophosphamide (AC) protocol has shown comparable efficacy with improved tolerability in unselected splenic HSA populations [4,11]. The protocol used in this study represents a further modification of the AC regimen with dose-adjusting and interval administration between both agents to enhance tolerability, yet its performance has not been assessed specifically in dogs with haemoperitoneum, a clinical context that may entail distinct perioperative risks and prognosis. Within the emergency context of haemoperitoneum, regimen selection and timing are critical considerations. Anthracycline–alkylator schedules are attractive when the clinical aim is to balance cytotoxic intensity with early tolerability, enabling treatment to commence within 1–2 weeks after splenectomy. Historical VAC regimens have been associated with higher rates of myelosuppression and gastrointestinal toxicity, which may be challenging in recently stabilised patients, whereas simplified doxorubicin–cyclophosphamide (AC) protocols have shown broadly comparable efficacy with improved safety profiles in unselected splenic HSA cohorts [3,4,9,11]. Moreover, emerging data suggest that timely initiation of adjuvant chemotherapy is associated with improved outcomes in non-metastatic splenic HSA [12], underscoring the pragmatic value of well-tolerated regimens that can be delivered without delay in haemodynamically patients.

Metronomic chemotherapy has been proposed as adjuvant or maintenance therapy in canine HSA, particularly in early-stage or metastatic disease. Although some reports suggest prolonged survival in selected patients, results remain inconsistent and vulnerable to time-dependent bias [13,14,15,16]. This bias arises when the interval before metronomic therapy begins is misclassified as ‘time on treatment’, thereby guaranteeing survival during that interval and inflating the apparent benefit (immortal-time bias). It can be mitigated by analytic strategies such as modelling treatment as a time-varying exposure or assigning groups at a predefined landmark time [17,18].

The aim of this retrospective study was to evaluate survival outcomes and clinical tolerability of a doxorubicin–cyclophosphamide (AC) protocol in dogs with histologically confirmed stage II splenic HSA presenting with haemoabdomen. This study also explored the potential association between maintenance metronomic therapy and long-term survival in a subset of patients.

## 2. Materials and Methods

### 2.1. Study Design and Setting

This was a retrospective case series conducted at a university veterinary teaching hospital between 2014 and 2025. Diagnosis of splenic HSA was confirmed by histopathology in all dogs. Cases were identified through electronic medical records and oncology/anaesthesia logs. Exclusion criteria were incomplete staging, insufficient clinical data for follow-up, or absence of histopathological confirmation.

### 2.2. Eligibility Criteria and Case Definitions

Dogs were eligible if they had stage II splenic HSA with concurrent haemoperitoneum (haemoabdomen) at presentation. Tumour rupture (free abdominal blood with a splenic mass) was the qualifying criterion for stage II in the absence of distant metastasis. Nodal status was recorded when available (N0–N2). Clinical stage was assigned according to established TNM frameworks [19,20]. All enrolled dogs fulfilled stage II with haemaperitonemum requirements (T2/ruptured mass, N0–N2, M0).

### 2.3. Pre-Operative Stabilisation and Staging

Pre-operative assessment included complete blood count, serum biochemistry, abdominal ultrasonography to characterise the splenic mass and screen for intra-abdominal spread, three-view thoracic radiographs to evaluate pulmonary nodules, and focused cardiac ultrasound (FAST-Echo) to screen for pericardial effusion or right atrial masses. Stabilisation measures (e.g., crystalloid/colloid resuscitation, packed red blood cell transfusion as required) were provided at clinician discretion prior to splenectomy. Baseline clinical and laboratory variables were recorded at presentation.

### 2.4. Surgical Procedure

All dogs underwent splenectomy. Intra-operative events (hypotension, transient arrhythmias, diffuse haemorrhage, adhesions requiring extended dissection) were abstracted from anaesthesia and surgical records and are reported descriptively; they were not graded using oncology toxicity scales. Post-operative surgical complications were retrieved from progress notes.

### 2.5. Chemotherapy Protocol (Anthracycline–Alkylator)

All dogs were offered adjuvant doxorubicin–cyclophosphamide (AC) every 21 days up to a protocol cap of five cycles. The regimen comprised doxorubicin 25–30 mg/m^2^ IV on day 1 and cyclophosphamide 250–300 mg/m^2^ PO on day 10 with furosemide concurrent administration (1 mg/kg single dose before cyclophosphamide) of each cycle. Dose selection was based on body-surface area, clinical status, and attending oncologist discretion. Chemotherapy commenced within 14 days of splenectomy in all cases. Adjuvant chemotherapy was targeted to commence within 14 days after splenectomy, contingent on clinical recovery and haematological fitness. Historically, when maintenance metronomic therapy was planned (pre-2019), the institutional schedule typically comprised three AC cycles; from 2019 onwards, five cycles were planned.

### 2.6. Maintenance Metronomic Therapy

A subset received maintenance metronomic therapy after completing the third or fifth AC cycle. Regimens included cyclophosphamide 12.5–15 mg/m^2^ PO q24h. In one dog, cyclophosphamide was replaced for chlorambucil (2 mg/m^2^/24 h) due to steril haemorragic cystitys as a cyclophosphamide adverse effect. Initiation of metronomic treatment occurred only after completion of the planned AC cycles.

### 2.7. Data Collection

Extracted variables included signalment, bodyweight, baseline clinicopathology, imaging findings, intra-operative events, transfusion support, staging details, the number and timing of AC cycles, dose adjustments, chemotherapy delays/omissions, toxicity, metronomic therapy, and follow-up status. Breed category (purebred vs. mixed-breed) and peri-operative variables were recorded to enable descriptive comparisons.

### 2.8. Outcomes

Overall survival (OS) was defined as time from splenectomy to death (all-cause) or last contact. Because clinical progression commonly preceded death by a short interval in this emergency presentation, disease-free interval (DFI) was not calculated. Tumour-specific survival (TSS) was not analysed because only one dog died of an unrelated cause. Dogs were censored if alive at study closure, lost to follow-up, or deceased due to non-HSA causes. Cause of death was determined by necropsy when available or inferred from consistent clinical/imaging evidence (e.g., metastases, haemoperitoneum, pericardial effusion) when necropsy was unavailable. Time to last follow-up (TLF) was recorded for all dogs.

### 2.9. Adverse Events and Grading

Chemotherapy-related toxicities were graded according to the Veterinary Cooperative Oncology Group—Common Terminology Criteria for Adverse Events, version 2 (VCOG-CTCAE v2; grades 1–5) [21]. Intra-operative anaesthetic/surgical events were not graded using VCOG-CTCAE and are presented descriptively.

### 2.10. Statistical Analysis

Descriptive statistics summarised baseline and treatment variables. Continuous data are reported as mean (SD) or median (IQR) according to distribution (Shapiro–Wilk test); categorical data as counts (percentages). Kaplan–Meier methods were used to estimate overall survival (OS), with log-rank tests for group comparisons. Univariable Cox models screened associations with OS; variables with *p* < 0.20 and judged clinically relevant were entered into multivariable modelling using backward stepwise selection.

To address survivor (immortal-time) bias, cumulative exposure to adjuvant doxorubicin–cyclophosphamide (AC) was encoded as a time-varying covariate that increased by one unit on the calendar date on which each cycle was administered (extended Cox models). In addition, fixed-time landmark analyses were performed at 60 and 92 days after splenectomy; dogs not surviving to a given landmark were excluded from the corresponding risk set. For metronomic therapy, a 120-day landmark was prespecified based on protocol timing; effect estimation was not attempted when complete separation occurred.

Proportional-hazards assumptions were evaluated graphically and using Schoenfeld residuals. Model discrimination was summarised with Harrell’s C-index. Two-sided *p*-values < 0.05 were considered statistically significant. Analyses were conducted in SPSS v30.0 (IBM Corp, International Business Machines Corporation; NY, USA) and Stata v15.0 (StataCorp LLC; TX, USA). These complementary time-aware approaches reduce, though cannot eliminate survivorship and selection biases inherent to retrospective cohorts [17,18].

## 3. Results

### 3.1. Baseline Cohort Characteristics

Twenty-one dogs with stage II splenic haemangiosarcoma and concurrent haemoabdomen were included. The cohort comprised 10 males and 11 females. Mixed-breed dogs represented 33.3% of cases, and most were neutered (66.7%). Baseline clinical and laboratory characteristics are summarised in Table 1 and Supplementary Table S1; numerical details are reported there to avoid redundancy in the text. When grouped as purebred versus mixed-breed, presentation did not differ materially; mixed-breed dogs were heavier (median 40.0 vs. 28.6 kg; *p* = 0.037).

Pre-operative haematology showed anaemia (haematocrit < 37%) in 68.4% and thrombocytopenia (<186 × 10^3^/µL) in 57.9% of cases. Median haematocrit was 34.7% (SD, 6.8). Additional laboratory findings are provided in Supplementary Table S1.

### 3.2. Peri-Operative Events and Treatment Delivery

Intra-operative complications were documented in 9/21 dogs (42.9%). These comprised hypotension in 6/21, transient anaesthetic arrhythmias (idioventricular rhythm, isolated premature beats or first-degree atrioventricular block, and/or bradycardia) in 4/21, diffuse intra-abdominal haemorrhage in 1/21, and splenic adhesions requiring extended dissection in 1/21; events were not mutually exclusive. No post-operative surgical complications were recorded. All dogs underwent splenectomy followed by a doxorubicin–cyclophosphamide (AC) protocol. The number of chemotherapy cycles ranged from 1 to 5 (median 3). Four dogs (19.0%) received maintenance metronomic chemotherapy after the third (*n* = 2) or fifth (*n* = 2) cycle. Initiation of adjuvant chemotherapy was not postponed; all dogs commenced AC within 14 days of splenectomy.

### 3.3. Chemotherapy-Related Adverse Events (VCOG-CTCAE v2)

Chemotherapy-related adverse events were uncommon and are summarised in Table 2; no treatment-related deaths occurred. Adverse events were graded according to VCOG-CTCAE v2.

### 3.4. Survival and Time-Aware Analyses

**Figure 1:**
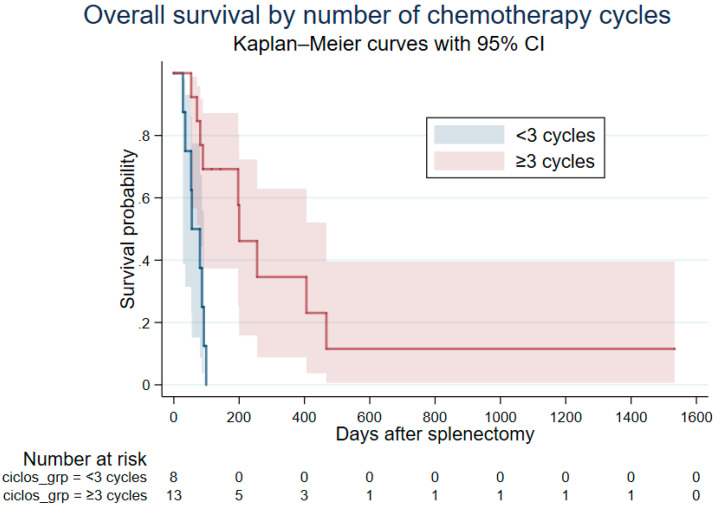
Overall survival in 21 dogs with splenic haemangiosarcoma and haemoabdomen treated with a doxorubicin–cyclophosphamide protocol, stratified by number of chemotherapy cycles (≥3 vs. <3). Dogs receiving ≥3 cycles (*n* = 13) showed significantly longer survival than those receiving <3 cycles (*n* = 8) (median 200 vs. 55 days, *p* = 0.004, log-rank test). Shaded areas indicate 95% confidence intervals. Numbers at risk are shown below the plot.

Of the 17 dogs that died, death was attributed to metastatic disease in 7 (41.2%), to recurrent haemoabdomen in 8 (47.1%), and to pericardial effusion in 1 (5.9%). One dog (5.9%) died from lymphoma, unrelated to haemangiosarcoma. Median overall survival (OS) was 92 days (95% CI: 72.6–111.4), with a range of 6 to 1533 days. The longest-surviving dog (>4 years; 1533 days) received adjuvant AC treatment followed by metronomic cyclophosphamide (12.5 mg/m^2^ PO q24h), later switched to chlorambucil (2 mg/m^2^ PO q24h) owing to sterile haemorrhagic cystitis due to cyclophosphamide administration. Dogs receiving ≥3 chemotherapy cycles had significantly longer OS than those receiving fewer (median: 200 vs. 55 days; HR: 0.16, 95% CI: 0.05–0.55; *p* = 0.004) (Figure 1). All dogs receiving metronomic therapy survived beyond the cohort median (median 467 days); given the very small subgroup and complete separation, these estimates are hypothesis-generating and should be interpreted with caution (Supplementary Figure S1; Table 3).

Across the baseline variables examined, none retained an independent association with overall survival after adjustment. Confidence intervals were wide and compatible with a range of effects; therefore, these results should be read as absence of evidence rather than evidence of absence. To address potential survivor bias, a time-varying Cox model showed a 34% reduction in the hazard of death per additional chemotherapy cycle (HR: 0.66, 95% CI: 0.50–0.88; *p* = 0.004). This association was supported by a 60-day landmark analysis (HR: 0.70, 95% CI: 0.52–0.94; *p* = 0.016); a 92-day landmark was under-powered (HR: 0.99, 95% CI: 0.36–2.71; *p* = 0.979). A 120-day landmark analysis for metronomic therapy could not be estimated because all treated dogs were long-term survivors. Time-dependent estimates are summarised in Supplementary Table S2.

## 4. Discussion

Regarding this spontaneous haemoabdomen, all dogs were treated with splenectomy followed by a standardised, doxorubicin–cyclophosphamide (AC) protocol. By focusing on a single anatomical location, defined clinical stage, and acute presentation, the study offers a precise assessment of the protocol’s tolerability and performance in a population often under-represented in published series. In keeping with this homogeneous design, clinical presentation did not differ materially between purebred and mixed-breed dogs; the latter were heavier, a finding that aligns with epidemiological observations in non-traumatic haemoperitoneum and splenic nodular disease where population-level breed over-representation does not translate into distinct presentation once haemoperitoneum is established [2,22]. In addition, favourable perioperative outcomes have been reported after splenic mass rupture, reinforcing that emergency presentation does not necessarily preclude safe surgical management or timely adjuvant therapy [23]. Use of antifibrinolytics such as tranexamic acid has been described in critically ill small-animal patients with neoplasia or haemorrhagic presentations, with generally mild adverse events [24].

Median overall survival (OS) in this series was 92 days (range, 6–1533), comparable to prior studies of splenic HSA treated with anthracycline-based adjuvant protocols [4,11,25], although these did not specifically isolate cases with haemoperitoneum. In contrast, series focusing on dogs with non-traumatic haemoabdomen and no systematic adjuvant treatment report shorter survival times, typically under three months [6,7]. Contemporary meta-analytic data indicate that >70% of dogs with non-traumatic haemoperitoneum due to a splenic mass harbour malignant disease [26], whereas large referral cohorts still report a substantial benign fraction, underscoring heterogeneity within emergency presentations [27,28]. While several patients in our series followed this trajectory, a subset achieved longer survival, including one dog surviving beyond four years. Adjuvant chemotherapy may be associated with improved prognosis; however, in the absence of a control group, this should be interpreted as associative rather than causal. Our findings align with reports indicating that AC-based adjuvant therapy can achieve outcomes comparable to VAC while improving tolerability [4,11]. Differences across series likely reflect case-mix, perioperative status (ruptured versus non-ruptured tumours), protocol scheduling (three cycles when maintenance was intended before 2019 versus five cycles thereafter), and the analytical treatment of time-dependent exposures. These therapeutic choices are also consistent with contemporary consensus recommendations [19]. Recent evidence suggesting that timely adjuvant initiation is associated with improved outcomes in non-metastatic splenic HSA [12] also accords with our observation that all dogs commenced adjuvant therapy within 14 days, without postponement and with low toxicity, supporting the feasibility of structured, early adjuvant management in haemoperitoneum.

The protocol used in this study omitted vincristine and adjusted doxorubicin and cyclophosphamide with the intent of improving tolerability and compliance. The treatment was well tolerated overall, with only one episode of grade 4 neutropenia and no dose-limiting toxicities or treatment-related deaths. These results compare favourably with historical VAC protocols, which have been associated with higher rates of haematological and gastrointestinal toxicity [3,9]. Our findings are consistent with prior work suggesting that AC-based regimens can offer similar efficacy with improved safety profiles relative to VAC [4,11]. Furthermore, emerging evidence supports the relevance of regimen choice and timing in non-metastatic splenic HSA [12,25], considerations that are particularly pertinent in clinically unstable dogs with haemoperitoneum. Diagnostic heterogeneity further complicates comparisons across cohorts; for example, ultrasonographic ‘cavitation’ has poor discriminatory value for HSA versus benign lesions [29]. The number of chemotherapy cycles completed was independently associated with OS. This association was supported by both landmark and time-varying Cox models, which we used to mitigate time-dependent (immortal-time) bias already outlined in the Introduction and Methods [17,18]. Although this variable has not consistently shown prognostic value in earlier series [11,25], differences in case mix, planned scheduling (three cycles when maintenance metronomic therapy was intended before 2019 vs. five cycles thereafter), statistical modelling, and adherence to treatment protocols may account for the discrepancy. Importantly, in our cohort receiving fewer cycles generally reflected early clinical deterioration and/or owner decision rather than toxicity-driven discontinuation, consistent with the low incidence of clinically significant adverse events.

Maintenance metronomic chemotherapy was associated with extended survival in four dogs, but interpretation is limited by the small sample size and complete separation in survival curves. This finding should be considered hypothesis-generating rather than conclusive. Similar trends have been reported in maintenance settings [15,30], whereas use of metronomic protocols as first-line therapy has not demonstrated significant benefit in early-stage disease [14]. Consistent with this, metronomic protocols have not demonstrated significant first-line benefit in early-stage disease [14,31], and time-dependent biases warrant cautious interpretation of maintenance effects. Prospective, time-aware evaluations will be required to clarify whether metronomic exposure confers incremental benefit beyond standard adjuvant protocols in this specific emergency presentation.

Pre-operative clinical or laboratory variables, including haematocrit, platelet count and total protein, were not independently associated with survival. This contrasts with earlier reports suggesting prognostic roles for baseline haematological parameters [1,25]. Our sample size and event count afforded limited power to detect modest prognostic effects, and the corresponding confidence intervals do not exclude the magnitude of associations previously reported for anaemia, thrombocytopenia, and haemoperitoneum in splenic haemangiosarcoma. These results should therefore be interpreted as absence of evidence rather than evidence of absence, underscoring the need for adequately powered prospective studies to refine risk stratification.

This study has limitations inherent in retrospective designs. The sample size was small, limiting statistical power and precision. Data were obtained from a single institution, and clinical decisions such as the initiation of metronomic therapy were not protocolised. Although we applied established methods to address time-dependent bias and reported toxicity using current standards (VCOG-CTCAE v2), residual confounding and information bias cannot be excluded. While time-varying and landmark approaches mitigate immortal-time bias, they do not eliminate residual confounding or selection effects intrinsic to retrospective cohorts [17,18]. These results should therefore be interpreted cautiously and regarded as exploratory. Future work should prospectively evaluate AC-based protocols potentially incorporating optimised timing [12] and predefined criteria for maintenance therapy in multi-centre settings to confirm generalisability and to determine whether specific subgroups (e.g., those achieving ≥3 cycles within 9–12 weeks) derive disproportionate benefit.

## 5. Conclusions

In conclusion, this case series supports the feasibility of a doxorubicin–cyclophosphamide protocol in dogs with stage II splenic HSA and spontaneous haemoabdomen. The protocol was well tolerated, with adverse events graded according to VCOG-CTCAE v2 and no treatment-related deaths, and longer survival was associated with the number of chemotherapy cycles completed in time-aware analyses. Given the retrospective design and absence of a control group, these observations should be interpreted as associative rather than causal. Despite inherent limitations, this study provides outcome data for a defined and clinically relevant subgroup and illustrates that timely adjuvant treatment (initiated within 14 days post-splenectomy) is practicable even after emergency presentation. Future work should prioritise prospective, multi-centre evaluation of AC-based protocols with predefined scheduling (such as three versus five cycles), incorporate prespecified, bias-mitigating analyses, and formally assess the role of maintenance metronomic therapy. Such studies will help refine risk stratification and guide evidence-based adjuvant strategies in dogs with ruptured splenic haemangiosarcoma.

## Figures and Tables

**Table 1:** Demographic and clinical characteristics of 21 dogs with stage II splenic haemangiosarcoma and haemoabdomen.

Variable	*n* (%)
Sex	
Male	10 (47.6%)
Female	11 (52.4%)
Neuter status	
Neutered	14 (66.7%)
Intact	7 (33.3%)
Breed (most common)	
Mixed breed	7 (33.3%)
Labrador Retriever	5 (23.8%)
German Shepherd	3 (14.3%)
Median age (years)	10.2 (8.7–12.0) *
Median weight (kg)	28.8 (24.8–37.2) *
Anaemia (Hct < 37%)	
Yes	13 (68.4%)
No	6 (31.6%)
Thrombocytopenia (<186 × 10^3^/µL)	
Yes	11 (57.9%)
No	8 (42.1%)
Intraoperative complications	
Yes	9 (42.9%)
No	12 (57.1%)
Postoperative complications	
Yes	0 (0%)
No	21 (100%)
Metronomic chemotherapy	
Yes	4 (19.0%)
No	17 (81.0%)

Table legend. The Asterix (*) indicated that data are expressed as median and interquartile range (IQR).

**Table 2:** Chemotherapy-related adverse events during adjuvant AC, graded by VCOG-CTCAE v2 [21] patient-level maximum grade across all cycles (*n* = 21 dogs).

System	Adverse Event	Grade 1 *n* (%)	Grade 2 *n* (%)	Grade 3–4 *n* (%)	Any Grade *n* (%)
Haematological	Neutropenia	1 (4.8)	0	1 (4.8)	2 (9.5)
Gastrointestinal	Diarrhoea	2 (9.5)	0	0	2 (9.5)
Constitutional	Anorexia/Inappetence	0	1 (4.8)	0	1 (4.8)
Summary	Dogs with ≥1 adverse event	—	—	1 (4.8)	5 (23.8)

Notes. Adverse events were graded by VCOG-CTCAE v2 [21]. Data are *n* (%) of dogs; highest grade per domain shown. GI, gastrointestinal. Intra-operative events were not VCOG-graded and are described in Section 3.2.

**Table 3:** Multivariable Cox model for overall survival (*n* = 21; deaths = 17).

Variable	Scale/Contrast	HR (95% CI)	*p*-Value
Chemotherapy cycles	Per additional cycle	0.60 (0.46–0.78)	<0.001
Metronomic therapy †	Yes vs. No	0.15 (0.03–0.78)	0.024

Model discrimination: Harrell’s C-index = 0.75. † Time-dependent exposure; interpret with caution in view of potential immortal-time bias. The apparent benefit was not estimable at the 120-day landmark (complete separation).

## Data Availability

The data presented in this study are available on request from the corresponding author, raw datasets are not publicly shared to protect client/patient privacy. Aggregated/derived data are provided within the article and its Supplementary Materials.

## Supplementary Materials

The following supporting information can be downloaded at https://www.mdpi.com/article/10.3390/vetsci12111053/s1, Figure S1: Kaplan–Meier curves for overall survival stratified by use of metronomic therapy; Table S1: Preoperative haematological and biochemical parameters (descriptive statistics); Table S2: Landmark and time-dependent Cox regression models evaluating the association between chemotherapy cycles and overall survival.

## Abbreviations

The following abbreviations are used in this manuscript:

HSA	Haemangiosarcoma
VAC	Vincristine–doxorubicin–cyclophosphamide
AC	Doxorubicin–cyclophosphamide
