# Supplementary material for: Doxorubicin–Cyclophosphamide Protocol in Dogs with Splenic Haemangiosarcoma and Haemoabdomen: A Retrospective Case Series
Source: Vet Sci. 2025 Nov 2;12(11):1053. doi: 10.3390/vetsci12111053 (PMC12661923; doi:10.3390/vetsci12111053)
Supplement: Supplementary file 1 [file vetsci-12-01053-s001.zip › vetsci-3917426-supplementary.pdf]

Supplementary Table S1. Preoperative haematological and biochemical parameters (descriptive statistics).

Variable	n	Mean ± SD	Median (IQR)	Range
Hct (%)	18	34.67 ± 6.79	–	23.1 – 48.5
Hb (g/dL)	19	10.92 ± 1.90	–	7.3 – 14.6
RBC (10^6/μL)	19	5.06 ± 0.98	–	3.36 – 6.67
MCV (fL)	18	63.34 ± 13.78	–	22.3 – 72.0
MCH (pg)	18	21.12 ± 2.00	–	16.5 – 23.9
MCHC (×10^3/μL)	18	31.48 ± 2.98	–	23.4 – 36.3
Platelets (×10^3/μL)	19	–	103.0 (87.0–186.0)	33 – 597
WBC (×10^3/μL)	18	–	15.25 (8.88–23.25)	3.36 – 35.98
Neutrophils (%)	17	–	17.84 (10.14–24.44)	5.23 – 31.92
Lymphocytes (%)	18	–	1.63 (1.12–2.10)	0.19 – 6.26
Monocytes (%)	18	–	0.90 (0.59–1.69)	0.02 – 3.70
Eosinophils (%)	17	–	0.10 (0.03–0.32)	0.00 – 1.60
Glucose (mg/dL)	18	–	114.57 (88.00–131.25)	64.47 – 287.0
BUN (mg/dL)	18	–	18.41 (9.79–29.52)	5.00 – 77.15
Creatinine (mg/dL)	18	–	1.13 (0.57–2.31)	0.57 – 2.31
Total proteins (g/dL)	17	–	8.02 (4.50–8.02)	4.50 – 8.02
ALT (U/L)	15	–	32.00 (23.00–44.48)	12.73 – 68.00

Hct = haematocrit; Hb = haemoglobin; RBC = red blood cells; MCV = mean corpuscular volume; MCH = mean corpuscular haemoglobin; MCCH = mean corpuscular haemoglobin concentration; BUN = blood urea nitrogen; ALT = alanine aminotransferase. Values are expressed as mean ± standard deviation (SD) for approximately normal distributions, and as median (interquartile range, IQR) for skewed distributions.

Supplementary Table S2. Landmark and time-dependent Cox regression models evaluating the association between chemotherapy cycles and overall survival.

Analysis approach	HR (95% CI)	p-value	N (events)	Interpretation
60-day landmark analysis (chemotherapy cycles)	0.70 (0.52–0.94)	0.016	16 (12)	30% risk reduction per additional cycle in dogs surviving beyond 60 days
Time-varying Cox model (chemotherapy cycles)	0.66 (0.50–0.88)	0.004	21 (17)	34% risk reduction per additional cycle, accounting for cumulative exposure
92-day landmark analysis (chemotherapy cycles)	0.99 (0.36–2.71)	0.979	9 (6)	No effect observed; underpowered due to small sample size
120-day landmark analysis (metronomic therapy)	Not estimable	–	7 (5)	Complete separation: all dogs receiving metronomic therapy were long-term survivors

Abbreviations: HR, hazard ratio; CI, confidence interval. Results reflect exploratory analyses addressing potential survivor bias using fixed-time landmark and time-dependent Cox regression approaches. "Not estimable" indicates model non-convergence due to complete separation.

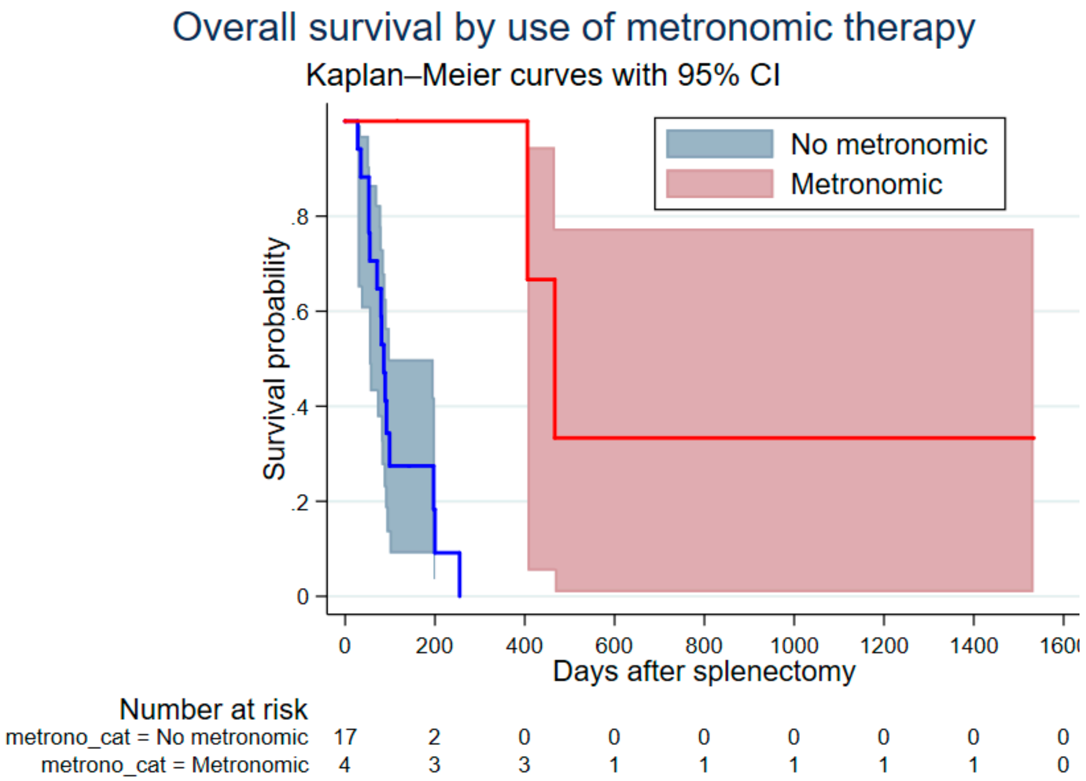

Supplementary Figure S1. Kaplan–Meier curves for overall survival stratified by use of metronomic therapy. Dogs receiving metronomic therapy after completion of the standard chemotherapy protocol (n = 4) showed prolonged survival compared to those who did not (n = 17). Median survival was not estimable in the metronomic group due to complete separation. Shaded areas indicate 95% confidence intervals. Risk tables are shown below the plot.
